# The evolution of infant-directed communication: Comparing vocal input across all great apes

**DOI:** 10.1126/sciadv.adt7718

**Published:** 2025-06-25

**Authors:** Franziska Wegdell, Caroline Fryns, Johanna Schick, Lara Nellissen, Marion Laporte, Martin Surbeck, Maria A. van Noordwijk, Shelly Masi, Birgit Hellwig, Erik P. Willems, Klaus Zuberbühler, Carel P. van Schaik, Sabine Stoll, Simon W. Townsend

**Affiliations:** ^1^ Department of Evolutionary Anthropology, University of Zurich, Zurich, Switzerland.; ^2^ Institute for the Interdisciplinary Study of Language Evolution, University of Zurich, Zurich, Switzerland.; ^3^ Department of Comparative Cognition, University of Neuchatel, Neuchatel, Switzerland.; ^4^ Ecoanthropologie, Centre National de la Recherche Scientifique/Muséum National d’Histoire Naturelle, University Paris Cité, Musée de l’Homme, Paris, France.; ^5^ Histoire naturelle de l’Homme préhistorique, UMR7194, PaleoFED, Muséum National d’Histoire Naturelle, Paris, France.; ^6^ Institut des Sciences du Calcul et des Données, Sorbonne Université, Paris, France.; ^7^ Department of Human Evolutionary Biology, Harvard University, Cambridge, MA, USA.; ^8^ Department of Human Behavior, Ecology and Culture, Max Planck Institute for Evolutionary Anthropology, Leipzig, Germany.; ^9^ Comparative Socioecology Group, Max Planck Institute of Animal Behavior, Konstanz, Germany.; ^10^ Department of Linguistics, University of Cologne, Cologne, Germany.; ^11^ School of Psychology and Neuroscience, University of St. Andrews, St. Andrews, UK.

## Abstract

Human language is unique among communication systems since many elements are learned and transmitted across generations. Previous research suggests that this process is best predicted by infant-directed communication, i.e., a mode of communication directed by caregivers to children. Despite its importance for language, whether infant-directed communication is unique to humans or rooted more deeply in the primate lineage remains unclear. To assess this, we investigated directed and surrounding vocal communication in human infants and infants of wild nonhuman great apes. Our findings reveal that human infants receive dramatically more infant-directed communication than nonhuman great ape infants. These data suggest that the earliest hominins likely relied more on surrounding communication to become communicatively competent, while infant-directed vocal communication became considerably more prominent with human language.

## Teaser

Human infants receive more directed communication than other great ape infants, indicating that it evolved alongside language.

## INTRODUCTION

Human language is unparalleled in its diversity, complexity, and informational potential. Through the production and combination of linguistic units, we can communicate about the past and the future, the real and the imagined. The acquisition of this unique communication system is highly dependent on linguistic input. One pivotal input type, infant-directed communication, is a crucial source of language learning and a key predictor of acquisition [e.g., (*1*–*3*)]. Infant-directed communication has been documented in many cultures and languages (*4*, *5*) and involves modifications to spoken language (*6*, *7*), sign language (*8*), as well as gestures (*9*) used when directly addressing a child or infant (hereafter infant). In the vocal domain, infant-directed communication is typically characterized by a number of acoustic (*5*, *10*) and structural (*11*–*13*) features and has been demonstrated to attract the infants’ attention more than adult-directed speech (*14*, *15*). These features have been shown to support language acquisition, both in comprehension (*3*, *16*) and production (*2*, *17*). A considerable body of research has further indicated that infant-directed communication plays a role in the transmission of cultural knowledge, a process commonly referred to as natural pedagogy (*18*). However, there is also substantial cross-cultural variation in infants’ exposure to infant-directed communication, with no obvious effect on language learning (*19*–*24*). As a result, recent studies have also begun to emphasize the potential augmentative role of infant-surrounding communication in language acquisition (*21*, *25*–*27*).

Despite the central role that infant-directed communication plays in language acquisition and cultural transmission, its evolutionary origins remain largely unknown (*28*). The few studies investigating the topic in our closest-living relatives, the nonhuman great apes, have suggested minimal (*29*) or no (*30*) infant-directed vocal communication, although targeted and systematic empirical studies of great ape individuals in their natural habitat are currently lacking. To reconstruct the evolutionary emergence of infant-directed vocal communication, we investigated the extent to which vocal communication is directed at human infants from different cultures (Chintang, Qaqet, Shipibo-Konibo, and Tuatschin) and infants from at least one species from each genus of all nonhuman great apes [Bornean orangutans (*Pongo pygmaeus wurmbii*), western gorillas (*Gorilla gorilla*), chimpanzees (*Pan troglodytes schweinfurthii*), and bonobos (*Panpaniscus*)] using comparable methods. On the basis of earlier work (*28*), we expected high levels of infant-directed vocal communication in humans and low levels in nonhuman great apes. On the other hand, we expected human and most nonhuman great ape infants to be exposed to a similar amount of surrounding communication (*28*).

To investigate differences in vocal input across species, we first compared the absolute amount of infant-directed and surrounding vocal communication (see Table 1 for definitions) between great ape species. In a second step, given underlying differences in the volubility of each species (see fig. S1), we compared the proportion of infant-directed and surrounding vocal communication in relation to the general vocal activity of each species. In all models, we also accounted for the infant’s age to control for age-related changes in infant-directed or surrounding communication. Our findings suggest that humans produce infant-directed vocal communication at levels that drastically exceed any other great ape species while the input from the surrounding environment across *Pan* species (chimpanzees and bonobos) and humans is broadly equivalent.

**Table 1.** Definition of vocal input types and vocal activity and summary of the data available per species. We counted the number of 2-min intervals in which a human observer recorded this type of vocal behavior. Infant-directed and surrounding vocal communication are independent of each other but can occur within the same 2-min interval. Abbreviations: H, humans; B, bonobos; C, chimpanzees; G, gorillas; OU, orangutans. See the Supplementary Materials for further details on the data availability for each species.

Type of vocal behavior	Definition	Species data availability
Infant-directed vocal communication	For nonhuman great apes, vocalizations were categorized as directed when, within the context of an ongoing dyadic interaction, the caller’s head was oriented 90° to 180° to the infant and the call led to an apparently satisfactory outcome (ASO, see table S2) (*68*). Our definition, which included both looking direction and an ASO, was maximally conservative and served to avoid including both false positive and false negative instances of infant-directed communication. For instance, in a joint travel initiation, a mother might look at an infant but only vocalize a few seconds later. On the other hand, long-distance calls can occur while individuals look around, also in the direction of the infant; however, these calls are not directed at the infant. When the caller’s head was not visible and it was possible that the caller was looking at the infant, we categorized their vocalization as uncertain. All data with uncertain vocalizations were subsequently excluded from the analysis. In the case of repeated calling, if the string of calls concluded in an ASO, they were categorized as directed. Laughter was always considered as a directed call as evidence shows that it is used to maintain play (*69*, *70*), and throat squeaks in orangutans were also always categorized as directed (*71*). For humans, annotations were based on linguistic context and visual information.	H, B, C, OU
Infant-directed vocal communication from mother	Directed vocalizations (as defined above) produced by the mother of the focal infant only.	H, B, C, G, OU
Infant-surrounding vocal communication	Vocalizations produced within audible proximity of the focal infant but not directed at them. For nonhuman great apes, long-distance vocalizations were always classed as surrounding vocal communication.	H, B, C, OU
Vocal activity	The overall volubility or “chattiness” of a species, including all audible vocalizations: surrounding, directed, and focal infant vocalizations.	H, B, C, G, OU

## RESULTS

To compare the total amount of (i) infant-directed and (ii) infant-surrounding vocal communication across species, we first fitted Bayesian generalized linear mixed-effects models (GLMMs), specifying a Poisson distribution (quantified by the number of 2-min intervals containing infant-directed or surrounding vocal communication per focal sample across species), see figs. S2 to S4 for visualizations of raw data. Focal samples were obtained by continuously following an immature individual (hereafter focal individual) for a set time period (*31*). To test whether a larger proportion of vocal communication is (i) infant-directed or (ii) surrounding (for visualizations of raw data of the vocal activity across species, see fig. S1), we secondly fitted Bayesian GLMMs, but this time specifying a binomial distribution. All analyses were controlled for differences in sampling effort [i.e., included the number of 2-min intervals per focal sample (on average 39.1 2-min intervals ±62.8) as an offset term in the GLMM] and accounted for overdispersion by incorporating an observation-level random effect.

### Species differences in infant-directed vocal communication

**Fig. 1.**
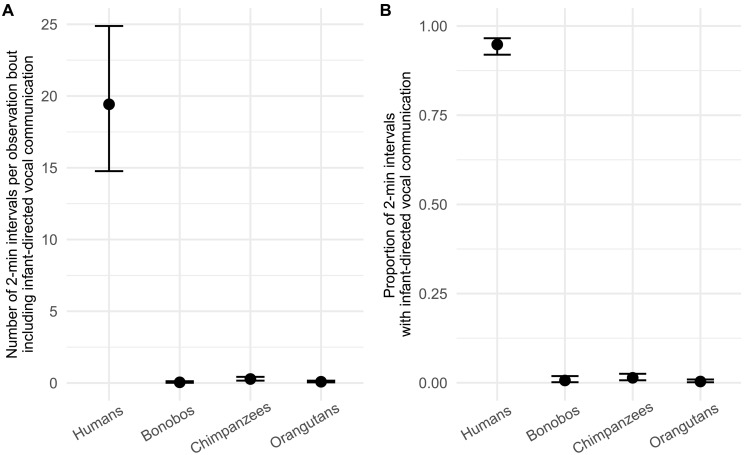
Species differences in infant-directed vocal communication. Model predictions showing the absolute amount of infant-directed vocal communication (**A**) and the proportion of infant-directed vocal communication relative to the general vocal activity (**B**) per species. The absolute amount of infant-directed vocal communication was calculated by the number of 2-min intervals (per focal observation bout) in which infant-directed vocal communication occurred. The relative amount of infant-directed vocal communication was calculated by dividing the number of 2-min intervals in which infant-directed vocal communication was reported, by the total number of 2-min intervals with any kind of vocalization. Model predictions from Bayesian GLMMs are calculated for each focal sample while controlling for differences in sampling effort. Error bars indicate 95% credible intervals.

Our first model yielded compelling support for the hypothesis that the incidence of infant-directed vocal communication is much higher in humans than in great ape species (Fig. 1A). Specifically, the number of 2-min intervals (per focal sample) in which infant-directed vocal communication occurred in humans [mean = 19.42, 95%, highest posterior density interval (HPD interval) = 14.62 to 24.66] was 399 times higher than in bonobos (mean = 0.05, 95% HPD interval = 0.01 to 0.11), 69 times higher than in chimpanzees (mean = 0.28, 95% HPD interval = 0.15 to 0.42), and 219 times higher than in orangutans (mean = 0.09, 95% HPD interval = 0.04–0.16). In addition, we found evidence for differences among the nonhuman great ape species. The number of 2-min intervals in which infant-directed vocal communication occurred in chimpanzees was three times higher compared to orangutans (95% HPD interval = 1.02 to 6.21) and five times higher compared to bonobos (95% HPD interval = 0.03 to 0.44). Moreover, a marginal positive effect of the focal individual’s age was identified, suggesting that in all species tested, as infants mature, they are incrementally exposed to more directed vocal communication [estimate: 0.02, 95% confidence interval (CI) = 0.01 to 0.04].

The second model then revealed that humans not only have higher absolute levels of infant-directed vocal communication but that infant-directed vocal communication forms a larger proportion of the vocal activity of humans (Fig. 1B). Specifically, we found that 94.8% of the vocal activity of humans (in the presence of an infant) consisted of infant-directed communication (95% HPD interval = 0.92 to 0.97), which was 144 times higher than in bonobos (prob. = 0.007, 95% HPD interval = 0.001 to 0.016), 66 times higher than in chimpanzees (prob. = 0.014, 95% HPD interval = 0.006 to 0.024), and 259 times higher than in orangutans (prob. = 0.004, 95% HPD interval = 0.001 to 0.008). We did not find any differences in the proportion of directed vocal communication between the nonhuman great ape species but we did again identify a small positive effect of the age of the focal individual, suggesting that older infants receive more directed vocal communication across development (estimate: 0.04, 95% CI = 0.02 to 0.07).

**Fig. 2.**
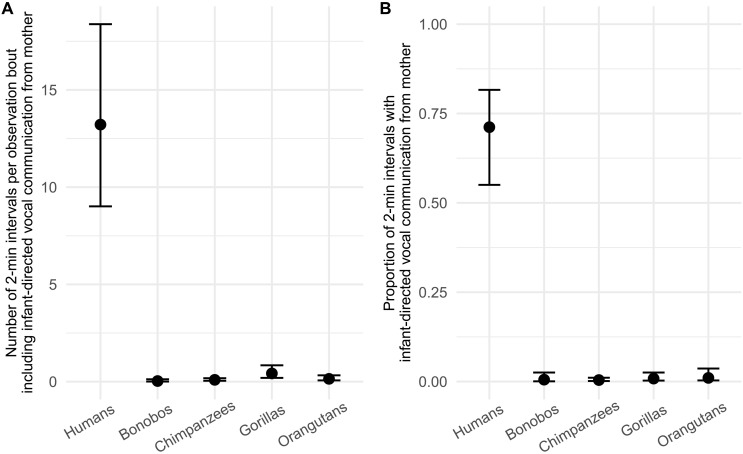
Species differences in infant-directed vocal communication from the mother. Model predictions showing the absolute amount of infant-directed vocal communication from the mother (**A**) and the proportion of infant-directed vocal communication from the mother relative to the general vocal activity (**B**) per species. The absolute amount of infant-directed vocal communication from the mother was calculated by the number of 2-min intervals (per focal observation bout) in which infant-directed vocal communication from the mother occurred. The relative amount of infant-directed vocal communication from the mother was calculated by dividing the number of 2-min intervals in which infant-directed vocal communication was reported, by the total number of 2-min intervals with any kind of vocalization. Model predictions from Bayesian GLMMs are calculated for each focal while controlling for differences in sampling effort. Error bars indicate 95% credible intervals.

When examining directed vocal communication from mothers only, our first model again yielded strong support that the incidence of infant-directed vocal communication is much higher in humans than in any of the other great ape species (Fig. 2A). Specifically, the number of 2-min intervals (per focal sample) in which infant-directed vocal communication from mothers occurred in humans (mean = 13.22, 95% HPD interval = 8.80 to 18.14) was 414 times higher than in bonobos (mean = 0.03, 95% HPD interval = 0.001 to 0.102), 143 times higher than in chimpanzees (mean = 0.09, 95% HPD interval = 0.037 to 0.166), 31 times higher than in gorillas (mean = 0.42, 95% HPD interval = 0.171 to 0.791), and 92 times higher than in orangutans (mean = 0.14, 95% HPD interval = 0.047 to 0.291). In addition, our model predicted marginal differences between the nonhuman great ape species. The number of 2-min intervals in which infant-directed vocal communication from mothers occurred in gorillas was three times higher compared to orangutans (95% HPD interval = 0.61 to 7.13), 13 times higher when contrasted to bonobos (95% HPD interval = 0.001 to 0.274), and five times higher when compared to chimpanzees (95% HPD interval = 0.05 to 0.509). For orangutans, the number of 2-min intervals in which infant-directed vocal communication from mothers occurred was five times higher compared to bonobos. We found no effect of the age of the focal individual on the amount of infant-directed vocal communication infants received from the mother.

The second model again indicated that, in humans, a greater proportion of the overall vocal activity is made up of directed communication from the mother compared to all other great ape species (Fig. 2B). Specifically, we found that 72% of the overall vocal activity of humans contains infant-directed vocal communication from the mother (95% HPD interval = 0.57 to 0.83), which was 134 times higher than for bonobos (prob. = 0.005, 95% HPD interval = 0.001 to 0.019), 170 times higher than for chimpanzees (prob. = 0.004, 95% HPD interval = 0.001 to 0.01), 86 times higher than for gorillas (prob. = 0.008, 95% HPD interval = 0.002 to 0.021), and 70 times higher compared to orangutans (prob. = 0.004, 95% HPD interval = 0.001 to 0.008). We did not find any differences in the proportion of directed vocal communication from the mother between the nonhuman great ape species.

### Species differences in surrounding vocal communication

**Fig. 3.**
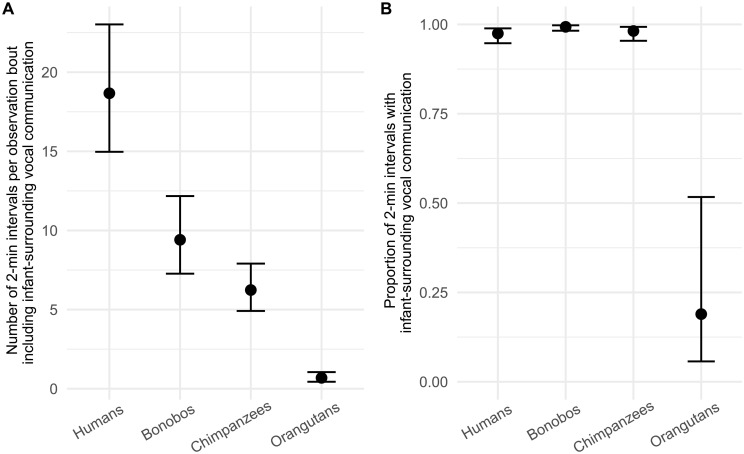
Species differences in infant-surrounding vocal communication. Model predictions showing the absolute amount of surrounding vocal communication (**A**) and the proportion of surrounding vocal communication relative to the general vocal activity (**B**) per species. The absolute amount of surrounding vocal communication was calculated by the number of 2-min intervals (per focal observation bout) in which surrounding vocal communication occurred. The relative amount of infant-directed vocal communication was calculated by dividing the number 2-min intervals in which infant-surrounding vocal communication was reported, by the total number of 2-min intervals with any kind of vocalization. Model predictions from Bayesian GLMMs are calculated for each focal sample while controlling for differences in sampling effort. Error bars indicate 95% credible intervals.

Our initial set of analyses demonstrated that humans engaged in more infant-directed communication, both absolutely and relatively when accounting for differences in vocal activity across species. Our next question was to understand how input from the surrounding vocal environment (in the presence of an infant) differed between species. Our first model yielded some support for the hypothesis that the incidence of infant-surrounding vocal communication is more similar between great ape species (Fig. 3A). Specifically, the number of 2-min intervals (per focal sample) in which infant-surrounding vocal communication occurred was highest in humans (mean = 18.67, 95% HPD interval = 14.84 to 22.86). More precisely, this was two times higher compared to bonobos (mean = 9.41, 95% HPD interval = 7.16 to 12.03), three times higher than for chimpanzees (mean = 6.24, 95% HPD interval = 4.82 to 7.80), and 27 times higher than the surrounding vocal input orangutan infants received (mean = 0.69, 95% HPD interval = 0.43 to 1.03). In addition, we found evidence for differences between the nonhuman great apes. The number of 2-min intervals in which infant-surrounding vocal communication occurred in bonobos was 1.51 times higher than in chimpanzees (95% HPD interval = 1.01 to 2.08) and 13.69 times higher than in orangutans (95% HPD interval = 5.16 to 14.42). Also, the number of 2-min intervals in which surrounding vocal communication occurred was 9.08 times higher in chimpanzees when compared to orangutans (95% HPD interval = 5.16 to 14.42).

Our second model predicted that infant-surrounding vocal communication occurred at proportionally similar levels in humans (mean = 97.5%; 95% HPD interval = 0.952 to 0.991) as for chimpanzee infants (mean = 98.2%; 95% HPD interval = 0.959 to 0.995). Bonobo infants were found to have a slightly higher (1.02) probability of receiving surrounding input than human infants (95% HPD interval = 0.04 to 0.74). In contrast, orangutan infants received five times less surrounding vocal communication (mean = 19%; 95% HPD interval = 0.04 to 0.46) compared to the other three great ape species (Fig. 3B). We found no effect of the age of the focal individual on the proportion of infant-surrounding vocal communication.

## DISCUSSION

Through comparing vocal input received by infants of all great ape species, we demonstrated notable differences in the amount of directed communication between human and nonhuman great apes. Humans engaged in infant-directed communication at orders of magnitude higher than any other great ape species. In contrast, we found fewer marked differences between species in terms of surrounding vocal input, with most nonhuman great apes displaying proportions similar to humans. Critically, our analyses showed that the vocal activity across species (i.e., how voluble a species is) was not sufficient to explain the differences between human and all nonhuman great apes.

A key implication of our data is that there must have been a massive expansion in the amount of infant-directed communication within the hominin lineage. What might explain this difference between humans and other great apes? Insights into the drivers of this expansion of infant-directed communication could be gleaned from our understanding of its function. One dominant hypothesis for the function of infant-directed vocal communication in humans is that these vocal interactions with children play a key role in scaffolding the transmission and learning of language. Our data provide compelling comparative support for this since nonhuman great ape vocal systems are generally considered to be far more fixed and genetically determined than humans’ (*32*), and, accordingly, we see much lower levels of infant-directed vocal input. However, humans not only direct vocalizations at infants but also adopt an idiosyncratic vocal register when doing so (e.g., repeating words and using higher pitch) [e.g., (*6*, *33*)]. Given the very low levels of infant-directed vocal communication in nonhuman great apes, it simply was not possible to also examine vocalizations for equivalent structural features known to characterize human infant–directed speech. To shed further light on any potential acoustic variation, in addition to better understanding the precise function of the rare infant-directed vocalizations in nonhuman great apes, behavioral data (e.g., the contexts in which these vocalizations are produced) compiled over longer study periods are critical. Previous research indicates that infant-directed gestures in great apes are, like infant-directed communication in humans, characterized by enhanced repetition (*34*, *35*) and might even be more frequent in nonhuman great apes in contrast to the low rates of infant-directed vocal communication (*29*, *36*, *37*). The gestural modality might therefore represent an additional fruitful avenue for future work investigating the evolutionary origins of infant-directed communication in humans.

Our focus here has been on the occurrence of infant-directed communication in our closest-living great ape relatives. Parallel research over the past 20 years has, however, also identified analogous or convergent cases in more distantly related species to humans. While informative, these cases of infant-directed vocal communication seem to serve qualitatively different functions than infant-directed vocal communication in humans. Functions range from infant-retrieval [e.g., domestic cats, *Felis catus*: (*38*)], mother recognition [e.g., Mexican free-tailed bats, *Tadarida brasiliensis*: (*39*)] to fine-tuning vocal production using vocal accommodation [e.g., orcas, *Orcinus orca*: (*40*)]. In marmoset monkeys (*Callithrix jacchus*) vocal input from caregivers has been shown to bootstrap infant vocal development. Specifically, contingent parental vocal feedback (within turn-taking events), but not the overall amount of surrounding parental vocalizations, had a positive effect on the development of adult-like vocalizations in immatures (*41*–*43*). Possible cases where the features of infant-directed vocal communication have parallels to human infant–directed vocal communication are found in greater sac-winged bats (*Saccopteryx bilineata*) as infant-directed vocal communication differs in pitch and timbre in comparison to adult-directed vocal communication (*44*). In addition, bottlenose dolphins (*Tursiops truncatus*) have also been shown to modulate the acoustic features of their signature whistles when their infant is present (*45*). Critically, both dolphins and greater sac-winged bats, in addition to humans, are considered vocal learners (*46*), highlighting a potential link between vocal learning and the presence of infant-directed vocal communication. Future studies investigating the presence and function of infant-directed vocal communication in vocal learning and nonvocal learning animals are required to support the generality of this relationship.

To better understand the overall vocal input infants are exposed to, we also captured the surrounding vocal communication of humans and other great apes. Our data indicate that infant-surrounding vocal communication is the major source of input in all nonhuman great ape species we tested. Our results also showed that orangutans received less surrounding vocal input compared to all the other great apes, including humans, a finding that can be explained by the fairly solitary nature of Bornean orangutans (*47*). Secondly, we found an additional, albeit much smaller, difference whereby bonobo infants received a marginally higher proportion of surrounding vocal input compared to humans (see Fig. 3). This difference can likely be explained by the greater amount of infant-directed communication in humans. An emerging picture from our data is that learning during vocal development in great apes must be nearly exclusively based on the surrounding (as opposed to directed) vocal input.

In some human cultures, infant-surrounding vocal input is also more prevalent than infant-directed vocal input (*21*–*23*), suggesting that surrounding vocal communication could also play a more important role for language acquisition than previously assumed. Such a conclusion is supported by growing evidence from more experimentally driven studies, demonstrating that children are not only able to learn language from surrounding interactions not explicitly directed toward them (*25*, *48*, *49*), but that the precise nature of the surrounding input can provide differential learning opportunities. For example, a recent study has indicated that, across cultures, surrounding speech from children captures the infants’ attention more effectively compared to surrounding speech from adults, suggesting that surrounding speech from other children might provide more learnable input compared to more complex adult speech (*50*). Following from this, a promising direction for further research would be to investigate the precise nature of infant-surrounding input for nonhuman great apes in greater detail—specifically focusing on the callers’ identities, age classes, relationship to the infant, and nature of the vocal input (whether, for example, it consists of calls or call combinations) and the potential influence this has on the infant’s vocal output.

In conclusion, our findings suggest that the tendency to direct vocalizations at infants, a key feature of human communication, has been massively expanded in the human lineage. These data provide support for the hypothesis that infant-directed vocal communication played a critical role in the emergence of human language through scaffolding the learning and acquisition of such a complex communication system. The presence of broadly equivalent levels of surrounding vocal communication in *Pan* (chimpanzees and bonobos) and humans suggests that early hominins probably relied on surrounding vocal communication for any learned component of their vocal system until infant-directed vocal communication became more prominent.

## MATERIALS AND METHODS

### Data

Vocal data on all species (Bornean orangutans, western gorillas, eastern chimpanzees, bonobos, and humans) were collected in a maximally comparable way. We collected data in wild-living populations for the nonhuman great apes and naturalistic conditions for humans. We continuously focal-followed (*31*) immatures (except for gorillas, where mothers were followed; see Material and Methods subsection “Gorillas”) of the same age range (10 to 60 months). This age range is adequate for a comparative study as all great apes have a similar life history with dependency on the mother lasting at least 4 years [chimpanzees and humans (*51*), western gorillas (*52*), bonobos (*53*) and orangutans (*54*–*56*)]. For all nonhuman great apes, we recorded all vocalizations uttered by the focal individual and any other individual that was audible (for exceptions and specifics for each species, see the methods description per species below). We noted if a vocalization was (a) directed toward an infant, and, if so, if it was directed by the mother or another individual, (b) part of surrounding vocal communication or (c) given by the focal individual (see Table 1 for detailed definitions). Vocalizations from unknown individuals were included in the analysis, provided that we could confidently exclude the mother or immature as the source and that we could determine whether the vocalization was directed or surrounding. If this was not the case, for gorillas, chimpanzees (C.F. dataset, see section “Chimpanzees”), and orangutans for which we conducted full-day focal follows, only the 2-min interval in which an unknown vocalization occurred was excluded. The human datasets did not include any 2-min intervals with uncertainty because of the high quality of both the video data and the linguistic transcriptions that provided sufficient information regarding the context of the interactions. The chimpanzee dataset (M.L.) also did not include any 2-min intervals with uncertainty because only focals with excellent visibility were used. For bonobos, if a vocalization was categorized as uncertain, the focal individual was recorded as out of view and we discarded the focal sample from that time onward. This difference in methodology is due to not conducting full-day focals for bonobos but focals of up to 1 hour. Our rationale for excluding all 2-min intervals following a 2-min interval with uncertainty was that uncertainty only arose when a vocalization occurred. If no vocalization occurred, the likelihood of uncertainty was zero. To avoid biasing our dataset through only excluding 2-min intervals with vocalizations, we decided to exclude all subsequent 2-min intervals from that focal observation. Although this might sound extreme, since we did not conduct full-day focal sampling for bonobos, this approach typically resulted in the exclusion of only a few minutes of data. While we appreciate that the approach for the bonobos is overly conservative and different to the other species’ data collection protocols, we were still keen to remove bias where we could, and that was only feasible with the bonobo dataset given the lengths of focals. Furthermore not excluding all subsequent 2-min intervals might have introduced some bias to the gorilla, orangutan, and chimpanzee (C.F.) data. However, since full-day focals were collected in these species, it simply did not make sense to exclude the rest of the day. Recording distances for all nonhuman great apes were between 7 and 15 m and for humans, approximately 2 to 10 m. To ensure that there was no intrinsic bias in how we coded our data, we conducted interobserver reliability testing using a blind coder for all human, chimpanzee (C.F. data), and bonobo datasets. Results suggest high levels of reliability in the assignment of vocalizations as either directed or surrounding (all Cohen‘s kappa values >0.8; see the Supplementary Materials). It is possible that recordings from humans tend to represent slightly higher rates of interactions compared to absolute naturalistic scenarios since non-daylong recordings typically only include situations where the infant is awake and surrounded by other individuals rather than being alone. Conversely, in nonhuman great ape species, there might be an underestimation of infant-directed vocal input due to the distance between observers and the individuals (minimum 7 m), which was even increased for chimpanzees and gorillas during the COVID-19 pandemic. Some great apes also tended to be in high and dense canopies, making it challenging to see the individuals and difficult to hear their calls. Hence, any data recorded when the individual was above 15 m and/or in such dense vegetation, were discarded. All data for this study were collected noninvasively and were purely observational. Informed consent was given by all caregivers of human infants. The data collection on nonhuman great apes adhered to the guidelines of the American Society of Primatologists for the ethical treatment of nonhuman primates.

### Bonobos

#### Study site and study groups

F.W. collected data from three wild bonobo communities at the Kokolopori Bonobo Reserve (N0.41716°, E22.97552°) (*57*) in the Democratic Republic of the Congo (DRC). At the time of the study, the Kokoalongo community consisted of 38 individuals (18 adults, 9 juveniles, and 11 infants), the Ekalakala community consisted of 21 individuals (12 adults, 3 juveniles, and 9 infants), and the Fekako community consisted of 9 individuals (6 adults, 1 juvenile, and 2 infants). Ethical permission to conduct the data collection was granted by the Institutional Animal Care and Use Committee at the Faculty of Arts and Sciences at Harvard University, the Institut Congolais pour la Conservation de la Nature (ICCN; 420/ICCN/DG-INT/03/12), the Ministry of Science and Technology of the DRC (MIN RST/SG/180/21; MIN RST/SG/180/23; MIN RST/SG/180/24) and is in line with the ethical guidelines of the former Department of Primatology at the Max Planck Institute for Evolutionary Anthropology.

#### Data collection

Data were collected from May to October 2022 from 05:30 a.m. to 05:00 p.m. using continuous focal follows of up to 60 min. If a focal individual went out of sight before 60 min, the minimum length for a focal sample to be included in this study was 6 min. Focal follows were conducted with a directional microphone [Sennheiser directional microphone (K6 power module, ME66 recording head and Rycote-Softie windscreen)] attached to a solid-state recorder (Marantz PMD 660). Vocalizations were recorded with a 44.1-kHz sampling frequency and a 16-bit amplitude resolution.

### Chimpanzees

#### Study site and study groups

C.F. and M.L. collected data in the Sonso community at the Budongo Forest Field Station (BCFS), Uganda (between 1°350 and 1°550 N and 31°080 and 31°420 E). The Sonso community (*58*) was composed of approximately 70 individuals (51 adults, 7 juveniles, and 17 infants in 2008 and 41 adults, 11 juveniles, and 11 infants in 2022). Ethics assessment was conducted, and permission for data collection was granted by the Uganda Wildlife Authority and the Uganda National Council for Science and Technology (UNCST-CF project registration number: NS272ES).

#### Data collection

M.L. collected data from January to December 2008 and by C.F. from February to September 2022. Data were collected from 07:00 a.m. to 04:30 p.m. except on Sundays where data were collected from 07:00 a.m. to 01:30 p.m. During the two study periods, 7 and 11 focal infants were followed, respectively. From the 1st to the 20th of February, additional COVID-19 protocols were in place and data could only be collected until 12.00 p.m. on any given day. From the 21st of February to the 6th of March, data could only be taken until 2.30 p.m. From the 7th of March onward, normal sampling days resumed. M.L. sampled continuously during 30- to 60-min (average of 51 ± 30 min) focals follows of an individual when visibility was not obstructed and then switched focal individuals throughout the day. M.L. sampled vocalizations in field notes and recorded vocalizations with an M66 Sennheiser directional microphone with the factory windshield and K6P power module. The microphone was attached to a solid-state recorder Marantz PMD660. Vocalizations were recorded with a 48-k sampling frequency and a 16-bit amplitude resolution. M.L. later merged and digitized the data. C.F. sampled continuously during full day follows and only switched focal individuals after 30 min of the focal individual being out of sight. C.F. recorded vocalizations with an M66 Sennheiser directional microphone with the factory windshield and K6P power module. The microphone was attached to a solid-state recorder Marantz PMD 661 MKII. Vocalizations were recorded with a 48-k sampling frequency and a 16-bit amplitude resolution. We compared the input rates between the two datasets and found them to be overall very similar. We merged the two datasets for chimpanzees for our analyses (for more detail, see the chimpanzee data comparison subsection in the Supplementary Materials).

### Gorillas

#### Study site and study groups

L.N. collected data from three groups of wild western gorillas in the Dzanga-Ndoki National Park of the Dzanga-Sangha Protected Areas in south-western Central African Republic (CAR). The CAR1 and CAR2 groups were studied in Bai Hokou (20°50′N, 16°28′E) and the CAR3 group in Mongambe (2°55′N, 16°23′E). The CAR1 group consisted of 8 individuals (4 adults, 2 juveniles and 2 infants); the CAR2 group consisted of 9 individuals (5 adults, 2 juveniles and 2 infants); and the CAR3 group consisted of 10 individuals (3 adults, 1 juvenile and 2 infants). Ethical evaluation and approval of the data collection was conducted by the Ethics Committee for Animal Experimentation - Cuvier Committee at the National Natural History Museum in France and the Ministre de la Recherche Scientifique et de l’Innovation Technologique of the Central African Republic (permit numbers: No. 020/MRSIT/DIRCAB/ CB.21 and No. 1PFGS21).

#### Data collection

Full-day follows on mothers with offspring were collected between 06:30 a.m. and 05:00 p.m., from April 2021 to March 2022. The 2-min intervals where the mother was in sight were summed up per day. This methodological difference, (i.e., focusing on the mother and not the infant) arose because the gorilla data were collected for a separate study. Data on vocalizations by all individuals, except laughter and play vocalizations, were recorded with a handheld Runbo device using cybertracker (see www.cybertracker.org). Although not recording laughter may have led to an underestimate of infant-directed vocalizations within the play context for gorillas, we did not record infant-directed laughter from the mother in any nonhuman great ape species, and for humans, no 2-min interval that was coded as “directed” included only directed laughter. Hence, despite these subtle differences, we are confident that our approach and results are still robust.

### Orangutans

#### Study site and study groups

C.F. collected data from February to June 2018 at the long-term field site of Tuanan, Mawas Reserve, Central Kalimantan, Indonesia (02° 15′’S; 114° 44′E). During this study period, 10 adult females and their dependent offspring and 15 adult males were regularly seen in the study area (*59*). The Indonesian State Ministry for Research and Technology (RISTEK, permit number: 02/EXT/SIP/FRP/E5/Dit.KI/I/2018), the Directorate General of Natural Resources and Ecosystem Conservation-Ministry of Environment Forestry of Indonesia (KSDAE-KLHK), the Ministry of Internal Affairs and the Nature Conservation Agency of Central Kalimantan (BKSDA KalTeng) gave their approval for this study.

#### Data collection

Data were collected from February to June 2018 from around 5 a.m. until the individuals built their evening nests. All vocalizations (including their call type and context) given and heard by the focal individual were noted continuously ad libitum throughout the focal sample. Mothers of each focal individual were simultaneously followed, and the simultaneous data collected from these follows were used to complete the vocal dataset, e.g., regarding the directedness of a vocalization. Duplicates of vocalizations were identified and removed from the dataset after data collection. Data were directly collected electronically on Excel with an iPad.

### Humans

All data from human infants were collected in their naturalistic language environments. Recording schemes differed between the different datasets from different languages and are described below. The minimum amount of data extracted from the different corpora per child per month was 60 min. Recording samples across languages were matched in age and gender as much as possible. Caregivers from all families gave their written (Qaqet, Shipibo-Konibo, and Tuatschin) or oral (Chintang) consent to participate in the data collection.

#### Shipibo-Konibo

J.S. collected the Shipibo-Konibo data between July 2021 and October 2022 in Callería, a small Shipibo-Konibo village in the Ucayali river valley. Shipibo-Konibo is a Panoan language spoken by approximately 20,000 people in the Ucayali river valley in the central eastern part of Peru (*60*). Data consist of naturalistic daylong video recordings in and around the homes of 14 children (11 to 54 months). One hour from each daylong recording was manually transcribed using the following sampling scheme: three segments of 10-min at 10:00 a.m., 01:00 p.m., and 04:00 p.m. Four randomly selected segments of 7.5-min before, between, and after the previously mentioned periodically selected clips. For each child, a total of 60 min of transcribed recordings from the same day were extracted from the corpus. The 4*7.5-min segments of each day were summed up to one 30-min segment for data processing. Ethical permission to conduct the data collection was granted by the University of Zurich and local representatives of the Shipibo-Konibo community.

#### Qaqet

B.H. collected the data for the Qaqet corpus between 2015 and 2016 in Raunsepna. Qaqet is a Papuan language spoken by approx. 15,000 people in Papua New Guinea’s East New Britain Province (*61*). Three children (age: 25, 32, and 41 months) were video-recorded within their environment by their parents or other adults for approximately 1 hour/ week. For this study, data from three following weeks of transcribed recording sessions from each child were extracted from the corpus resulting in a total of 2 hours of recordings per child on average. Ethical permission to conduct the data collection was granted by the La Trobe University, the National Research Institute of Papua New Guinea, the local NGO Bainings Environmental Heritage Conservation Foundation Inc., and the elders and representatives of the communities.

#### Chintang

The Chintang Language Research Program collected the data we used of the Chintang corpus in 2004 in Chintang, a village located in the lower foothills of the Himalayas (Dhankuṭā district) in Nepal. Chintang is a Sino-Tibetan language spoken by approximately 5000 speakers in the Himalayas (Kiranti group) (*62*). Children were video-recorded for a total of 4 hours during several sessions within a single week. For this study, we extracted 1 hour of transcribed recordings per child (age: 26, 32, 41, and 46 months) from the corpus from a total of four children.

#### Tuatschin

G. Walther and J. Mazara collected the data for the Tuatschin corpus between 2016 and 2019 in the Val Tujetsch as well as with Sursilvan-Tuatschin–speaking families in the Swiss diaspora. Tuatschin is a dialect of the Romansh Sursilvanvariety spoken by approximately 1500 speakers in the Val Tujetsch area (Switzerland). Children were video-recorded for a total of at least 4.5 hours, several times a week without a researcher being present. For this study, 100 min of transcribed recording sessions per child (age: 26, 28, 32, 37, 42, and 46 months) were extracted from the corpus from a total of six children. Ethical permission to conduct the data collection was granted by the University of Zurich.

### Data annotation

We manually annotated data from all five species to estimate the rates of directed and surrounding communicative input infants receive. All recordings were analyzed using zero-one sampling (*31*) in 2-min intervals. For the bonobo, chimpanzee, orangutan, and human data, every 2-min interval was coded for the presence or absence of infant-directed vocal communication, infant-directed vocal communication from the mother only, infant-surrounding communication, or vocal activity (see Table 1 for definitions). There could be overlap in inputs with a given 2-min interval containing multiple types of input. If that was the case, the interval was taken into account for each separate category. For the gorilla data, only infant-directed vocal communication and vocal activity could be annotated. General vocal activity was computed by adding together all 2-min intervals within one recording (or, in the case of gorillas: 1 day) in which either a surrounding, directed, or an infant vocalization occurred. For humans, prelinguistic vocalizations and laughing were included in the annotations given that they also have valuable communicative functions.

### Statistical analysis

To investigate species (bonobo, chimpanzee, gorilla, orangutan, and human) differences in infant-directed and infant surrounding (no data available for gorillas) vocal communication, in a first step, we formulated three Bayesian GLMMs, specifying a Poisson distribution. The respective outcome variables of these models were the number of 2-min intervals (per focal sample) with any (i) infant-directed vocal communication, (ii) infant-directed vocal communication by the mother, and (iii) infant-surrounding vocal communication. To answer the question whether species differed in relative terms, in a second step, we investigated differences in the proportion of infant-directed and infant-surrounding vocal communication to vocal activity. Specifically, we explored potential differences across species by dividing the number of 2-min intervals in which infant-directed vocal communication was reported, by the total number of 2-min intervals with any kind of vocalization. We used Bayesian binomial GLMMs to control for the vocal activity of each species. The respective outcome variables of these models were: (i) the proportion of all vocal inputs scored as infant-directed vocal communication, (ii) the proportion of all vocal communication scored as infant-directed vocal communication produced by the mother, and (iii) the proportion of all vocal communication scored as surrounding.

Each model incorporated species and infant age as potential explanatory variables while accounting for differences in sampling effort by including an offset term (the natural logarithm of the number of 2-min intervals that made up each focal sample). Random intercepts were estimated to account for multiple observations on each focal individual (infant ID), as well as the observed over-dispersion [i.e., an Observation Level Random Effect (*63*)].

We fitted our models in R (*64*) (version 4.3.2), using the “brms” (*65*) interface to Stan (*66*). Parameters were estimated by running four independent Monte Carlo Markov chains for 8000 iterations each (6000 to warm-up and 2000 to sample the posterior distribution). To facilitate model convergence, we specified weakly regularizing priors. Chain convergence, mixture, and stationarity were confirmed by visual inspection of trace plots and by ensuring that all $\hat{R}=1.00$ . To achieve this, the adapt_delta-argument was increased to 0.98.

Overall model performance was assessed by performing visual posterior predictive checks and by calculating a Bayesian $R^{2}$-statistic (*67*). Post hoc contrasts were calculated to quantify pairwise species differences. The outcome of all analyses can be found in the Supplementary Materials (see tables S3 to S8).

The respective human language could not be included as a variable to the main model since it would require lumping all nonhuman species into a single “nonlinguistic” category. We therefore conducted an additional within-species analysis with the four human datasets, as well as with the two chimpanzee datasets that were added together. Results of both comparisons can be found in the Supplementary Materials.

## Supplementary Materials

### This PDF file includes:

Supplementary Text Figs. S1 to S9 Tables S1 to S8 References
