# Supplementary material for: The evolution of infant-directed communication: Comparing vocal input across all great apes
Source: Sci Adv. 2025 Jun 25;11(26):eadt7718. doi: 10.1126/sciadv.adt7718 (PMC12190005; doi:10.1126/sciadv.adt7718)
Supplement: Supplementary file 1 — Supplementary Text Figs. S1 to S9 Tables S1 to S8 References [file sciadv.adt7718_sm.pdf]

Science Advances
AAAS

Supplementary Materials for

The evolution of infant-directed communication: Comparing vocal input across all great apes

Franziska Wegdell *et al.*

Corresponding author: Franziska Wegdell, franziska.wegdell@iea.uzh.ch; Caroline Fryns, caroline.fryns@unine.ch; Johanna Schick, johanna.schick@uzh.ch

Sci. Adv. **11**, eadt7718 (2025)
DOI: 10.1126/sciadv.adt7718

This PDF file includes:

Supplementary Text
Figs. S1 to S9
Tables S1 to S8
References

Supplementary Text

Inter-observer reliability (IOR)

We conducted IOR on the classification of vocalisations (infant-directed or infant-surrounding) in bonobos and chimpanzees where supplementary video data were available. For bonobos and chimpanzees, we could extract 19 and 20 video clips, respectively, in which infants heard a vocalisation. An independent individual then classified these calls as either infant-directed or infant-surrounding. Cohen's Kappa scores were calculated using the 'irr' package (*72*) and results indicated an excellent combined agreement between observers of 0.915 (Cohen's Kappa for Bonobos: 0.83 and for chimpanzees: 1) (*73*). Since no videos were collected for gorillas and orangutans, and there was always only one researcher present at each field site (due to logistical and financial reasons), we could not assess observer reliability directly. Nevertheless, we are confident our data has been collected objectively and in an unbiased way for the following reasons. Firstly, all coders discussed the definitions of infant-directed and surrounding communication before and after data collection at length, maximising consistency in call annotation across observers. Second, two data sets (chimpanzee 2022 data and orangutan data) were collected by the same researcher (CF) thereby excluding between-observer differences for these species. Third, we analysed two independent data sets and comparisons of the input indicated the sampled rates were similar between the two researchers that collected the chimpanzee data sets, CF and ML, despite the fact they were collected 10 years apart, arguably representing an indirect test of reliability (see chimpanzee data set comparison in the Supplementary Material).

For the human data, a naïve second coder double-coded a randomly selected 15% of each language. For all four data sets, inter-rater reliability was high (Cohen's Kappa: Tuatschin = 0.95; Shipibo-Konibo = 0.89; Qaqet = 0.88; Chintang = 0.93), indicating excellent agreement.

Human data comparison

In order to explore cultural differences within the human data set, we fitted three Bayesian Generalized linear mixed models (GLMMs) to compare directed and surrounding input across the four cultures. We fitted Bayesian GLMMs with the same model settings as in the main, cross-species analyses and used input type as the dependent variable with language and age of the focal individual

as fixed effects and focal individual ID and running number as random effects. We used post-hoc contrasts to compare pairwise differences in input rates.

Results When comparing the total directed input infants receive across cultures, our model indicated that there are no cultural differences (see Figure S5A). The model further predicted an effect of age of the focal infant (Estimate: 0.06, 95% CI = [0.01, 0.11]), suggesting that as human infants grow older, they receive more directed input (see Figure S5B).

In a next step, we compared the directed input infants receive across cultures only from mothers. Our model indicated that the probability for Chintang, Qaqet and Shipibo-Konibo infants receiving directed input from mothers is lower compared to Tuatschin infants (Chintang: odds.ratio = 0.20, 95% HPD = [0.01, 0.87]; Qaqet: odds.ratio = 0.04, 95% HPD = [0.001, 0.25]; Shipibo-Konibo: odds.ratio = 0.16, 95% HPD = [0.001, 0.60]) (see Figure S6A).

Finally, when comparing the surrounding input infants receive across cultures, our model indicated higher probabilities for Shipibo-Konibo and Chintang infants to receive surrounding input when contrasted with Tuatschin infants (Shipibo-Konibo: odds.ratio = 57.7, 95% HPD = [5.61, 195.93]; Chintang: odds.ratio = 84.14, 95% HPD = [4.25, 351.68]) (see Figure S6B).

Chimpanzee data set

The chimpanzee data set included data collected within two different time periods: data was collected in the year 2008 by ML and in the year 2022 by CF. Before merging both data sets we tested for comparability through contrasting the amounts of infant-directed and surrounding communication in both data sets. To this end, we fitted three binomial Bayesian GLMMs with the same model settings as in the main, cross-species analyses. These models used the proportion of input type (surrounding, directed and directed from mothers) of the general vocal activity of the community as the response variable but, instead of species, observer identity was used as the independent variable. We then used post-hoc contrasts to compare pairwise differences.

Results The directed model suggested no difference between observers and estimated 1.9% (CI=95%, HPD = [0.006, 0.04]) of directed input out of the vocal activity of the community in 2022 compared to 0.3% (CI=95%, HPD = [0, 0.007]) in 2008 (see Figure S7). The directed only from mothers model found no difference between observers and estimated 0.5% (CI=95%, HPD = [0.002, 0.01]) of directed input out of the vocal activity of the community in 2022 compared to 0.4% (CI=95%, HPD = [0, 0.01]) in 2008 (see Figure S8). The surrounding input model found a marginal difference between observers and estimated the data had 93% (CI=95%, HPD = [0.85, 0.98]) of surrounding input out of the vocal activity of the community in 2022 compared to 98% (CI=95%, HPD = [0.96, 0.99]) in 2008 which corresponds to 1.05 times more surrounding vocal communication in ML's data set (see Figure S9).

These results suggest no difference in the rates of infant-directed vocal communication across both chimpanzee data sets. Results further suggest a subtle variation in the rates of infant-surrounding vocal communication. Given the comparable rates of infant-directed vocal communication across data sets, the variation in the surrounding vocal communication can likely be explained by between study period differences in the community social dynamics. For these reasons, we concluded that the data sets could be merged and used as one.

Supplementary Figures

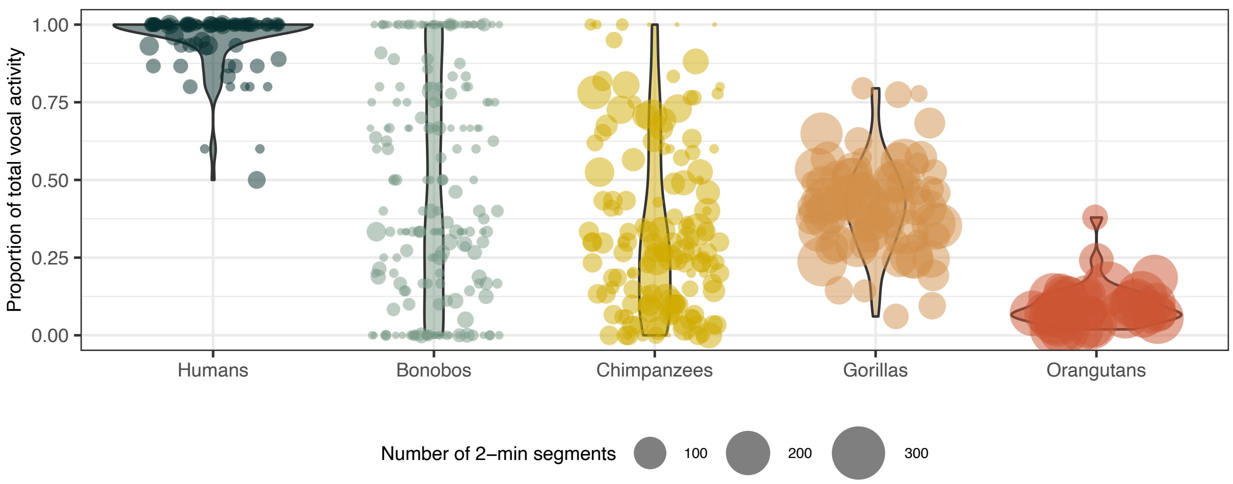

Figure S1: **Visualisation of the raw data: Proportion of total vocal activity per focal sample across species.** Each data point represents a focal sample. The size of the data point represents the number of two-minute segments each focal sample consists of.

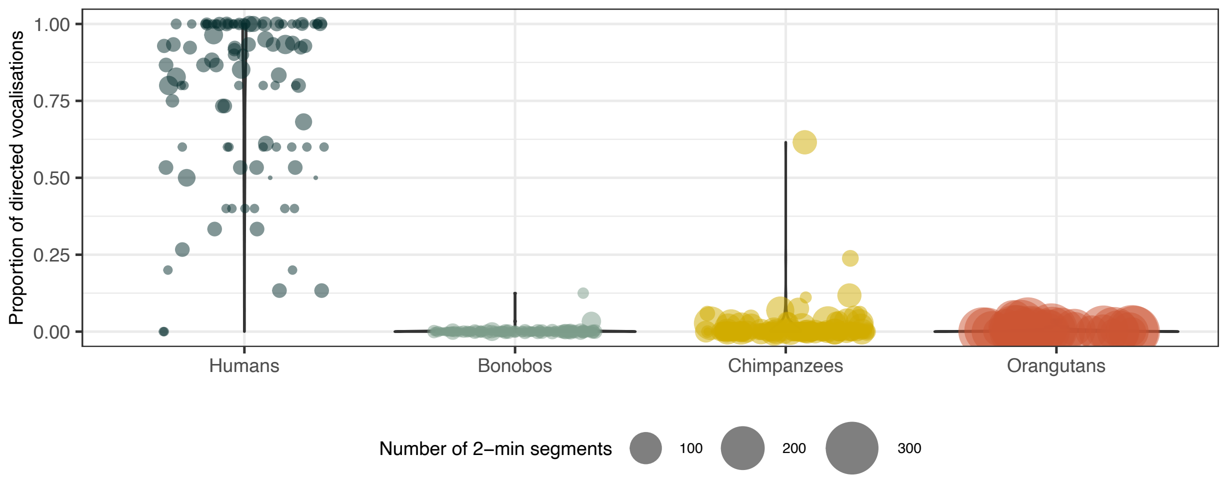

Figure S2: **Visualisation of the raw data: Proportion of directed vocalisations per focal sample across species.** Each data point represents a focal sample. The size of the data point represents the number of two-minute segments each focal sample consists of.

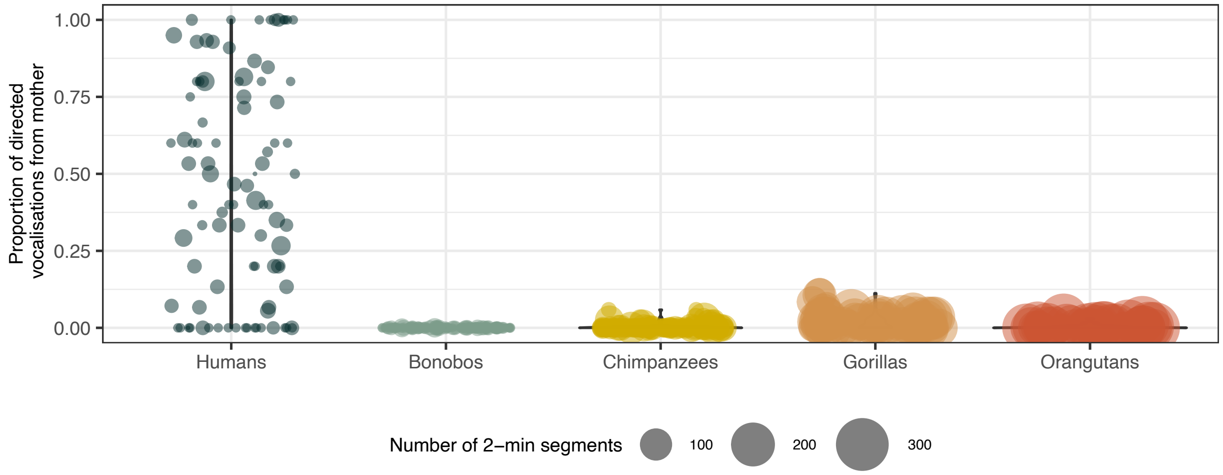

Figure S3: **Visualisation of the raw data: Proportion of directed vocalisations from mothers per focal sample across species.** Each data point represents a focal sample. The size of the data point represents the number of two-minute segments each focal sample consists of.

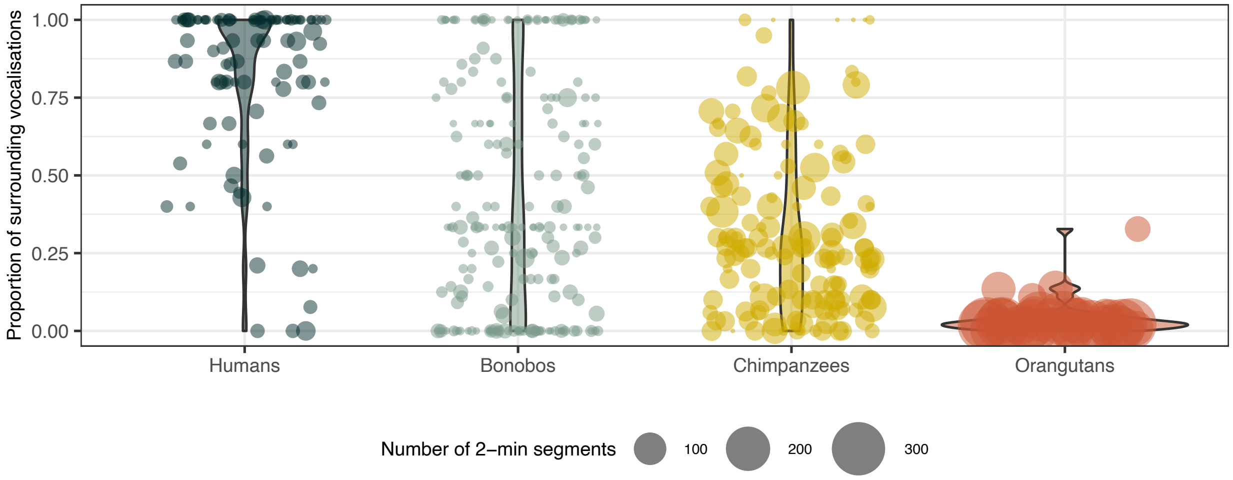

Figure S4: **Visualisation of the raw data: Proportion of surrounding vocalisations per focal sample across species.** Each data point represents a focal sample. The size of the data point represents the number of two-minute segments each focal sample consists of.

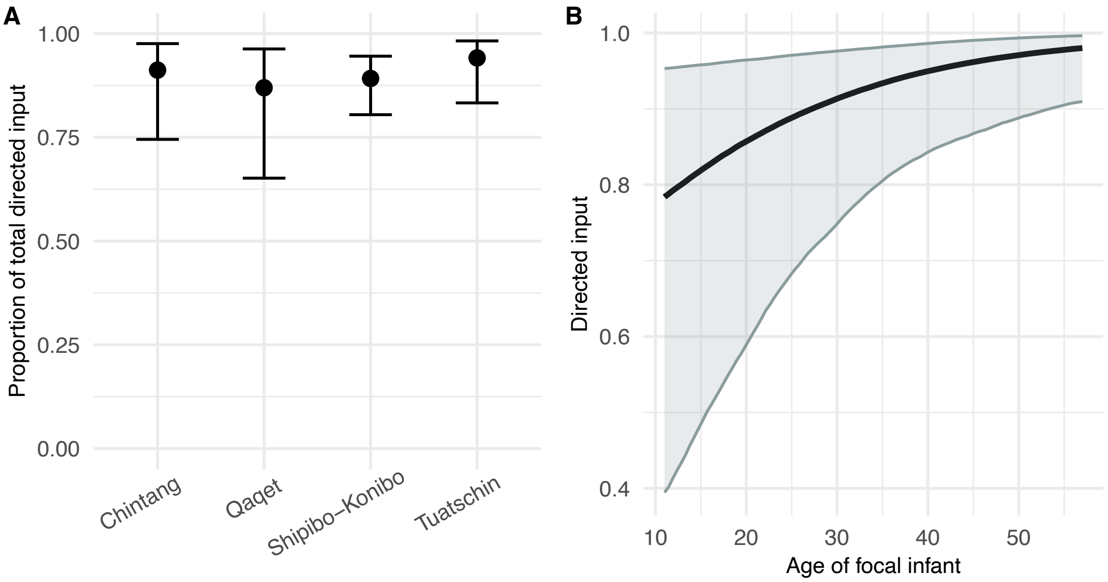

Figure S5: **Comparison between human cultures: Model predictions of overall directed input across cultures (A) and directed input as a function of age of focal infant (B).** Error bars and shaded areas indicate 95% credible intervals.

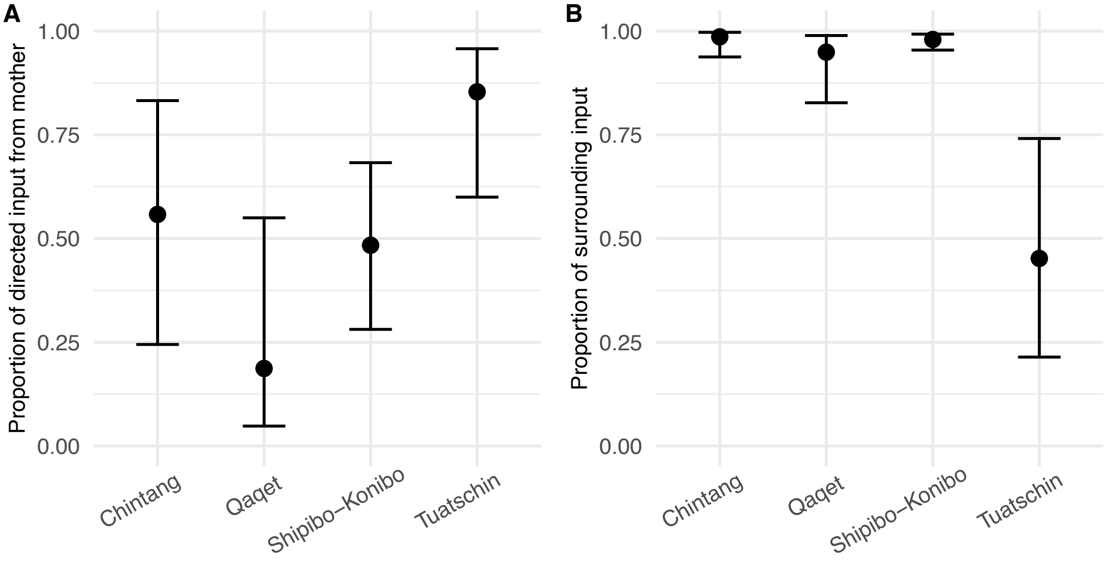

Figure S6: **Comparison between human cultures: Model predictions of directed input from mothers across cultures (A) and surrounding input across cultures (B).** Error bars indicate 95% credible intervals.

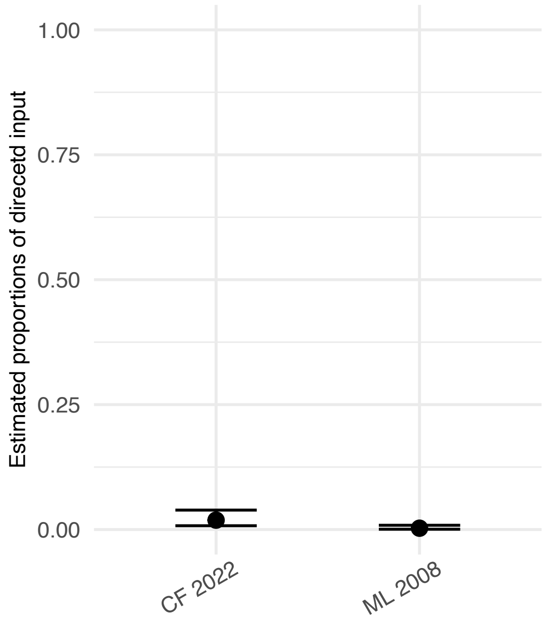

Figure S7: **Chimpanzee data set comparison: Model predictions of directed input across different observer data sets.** Predictions obtained from a Bayesian GLMM specifying a binomial distribution. Error bars indicate 95% credible intervals.

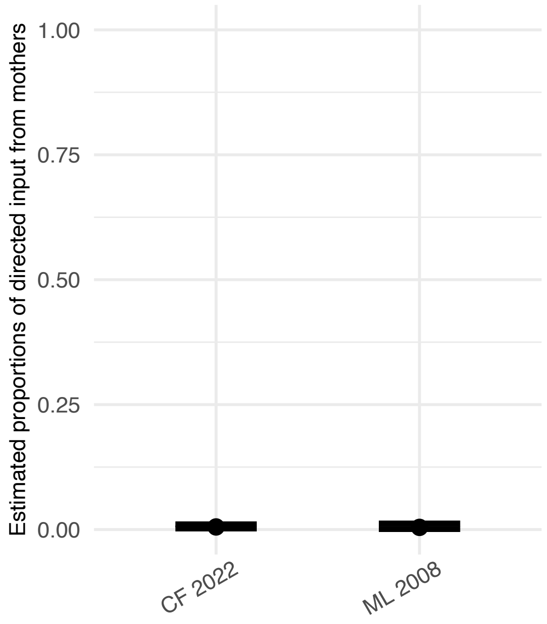

Figure S8: **Chimpanzee data set comparison: Model predictions of directed input from mothers across different observer data sets.** Predictions obtained from a Bayesian GLMM specifying a binomial distribution. Error bars indicate 95% credible intervals.

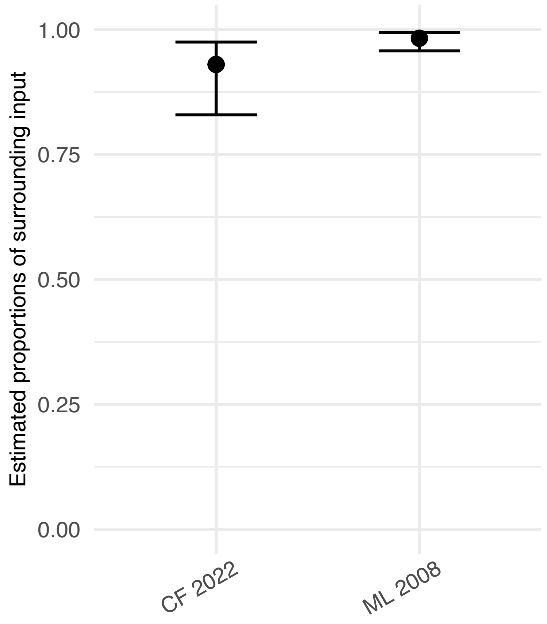

Figure S9: **Chimpanzee data set comparison: Model predictions of surrounding input across different observer data sets.** Predictions obtained from a Bayesian GLMM specifying a binomial distribution. Error bars indicate 95% credible intervals.

Supplementary Tables

Species	Number of infants	Age (months)	Average duration of focal sample (min)	Total observation time (hrs)
Bonobo	15	10-15, 33-48	12.7 ± 8.81	46.1
Chimpanzee	18	12-28, 30, 31, 35-38, 48-50, 52, 54, 55, 57-60	58.7 ± 41.9	142.9
Gorilla	7	11-16, 29-39, 41-46	244 ± 138	496.6
Orangutan	6	21, 23, 27, 39, 40, 42, 43, 46, 47, 56, 59	381 ± 149	228.7
Human	3 (Qaqet)	25, 30, 41	28.1 ± 14.2	6.6
	4 (Chintang)	27, 40, 41, 46	25.8 ± 14.7	5.2
	6 (Tuatschin)	26, 28, 32, 37, 42, 46	34.7 ± 12.4	9.8
	14 (Shipibo-Konibo)	11, 12, 13, 13, 15, 23, 23, 24, 26, 30, 34, 35, 41, 47	14.8 ± 8.5	14

Table S1: **Overview of number and age of focal infants and focal sample duration and observation time across species.** Data for the gorillas differ slightly for the infant-directed vocal communication from the mother. One bonobo infant is an orphan and could not be included in the "directed input from mother" analysis.

ASO	Definition	Example
Approach	The infant comes closer to the caller.	In a travel initiation context, the mother would call and start traveling. The infant would follow suit.
Reply vocally	The infant produces a call	In a travel context, the mother calls to initiate travel, and the infant replies
Stop action	The infant stops the behaviour it was exhibiting.	In a feeding context, the infant tries to take the food item from the same patch as another individual. The individual vocalises, and the infant does not take the food item.

Table S2: **Description of Apparently Satisfactory Outcomes (ASO).** ASOs have been used to establish the goal and intention of the caller (*68*). We adopted this approach to facilitate and support the definition of directed vocalisations. We used three broad ASOs - approach, reply vocally and stop action - to accommodate the range of variations in behaviours across species. All ASOs needed to happen within 10 seconds of the call. The ASOs for chimpanzee (data set CF) and gorilla data set were taken in real time. The ASOs for the orangutan and the second chimpanzee data set (data set ML) were taken post-hoc from field notes that were taken in real time. These notes included behavioural responses to vocalisations.

Contrast	Odds Ratio	Lower HPD	Upper HPD
H / B	398.90	98.49	1157.76
H / C	69.36	39.64	115.09
H / OU	219.16	92.49	405.95
B / C	0.18	0.03	0.44
B / OU	0.54	0.06	1.46
C / OU	3.14	1.02	6.21

Table S3: **Model results of post-hoc contrasts of pairwise differences of total directed input across species.** H=humans, B=bonobos,C=chimpanzees, OU=orangutans. HPD interval probability: 0.95.

Contrast	Odds Ratio	Lower HPD	Upper HPD
H / B	2814.92	491.66	8921.34
H / C	1298.34	537.59	2581.78
H / OU	5182.63	986.96	12141.94
B / C	0.46	0.05	1.36
B / OU	1.79	0.09	5.89
C / OU	4.01	0.67	9.88

Table S4: **Model results of post-hoc contrasts of pairwise differences of total directed input across species, controlled for vocal activity.** H=humans, B=bonobos,C=chimpanzees, OU=orangutans. HPD interval probability: 0.95.

Contrast	Odds ratio	Lower HPD	Upper HPD
H / B	413.76	53.37	2020.28
H / C	142.92	54.09	277.19
H / G	31.31	11.37	62.39
H / OU	91.95	24.07	189.95
B / C	0.34	0.013	1.27
B / G	0.08	0.001	0.27
B / OU	0.22	0.003	0.83
C / G	0.22	0.053	0.51
C / OU	0.64	0.14	1.58
G / OU	2.94	0.61	7.13

Table S5: **Model results of post-hoc contrasts of pairwise differences of directed input from mothers across species.** H=humans, B=bonobos, G=gorillas, C=chimpanzees, OU=orangutans. HPD interval = 0.95.

Contrast	Odds ratio	Lower HPD	Upper HPD
H / B	482.496	27.20	2430.39
H / C	590.34	104.00	1556.12
H / G	296.58	40.30	850.36
H / OU	234.58	17.40	750.35
B / C	1.23	0.005	6.03
B / G	0.62	0.001	3.00
B / OU	0.49	0.002	2.70
C / G	0.50	0.04	1.76
C / OU	0.40	0.03	1.50
G / OU	0.80	0.04	3.18

Table S6: **Model results of post-hoc contrasts of pairwise differences of directed input from mothers across species, controlled for vocal activity.** H=humans, B=bonobos, G=gorillas, C=chimpanzees, OU=orangutans. HPD interval = 0.95.

Contrast	Odds Ratio	Lower HPD	Upper HPD
H / B	1.97	1.41	2.75
H / C	2.99	2.08	3.96
H / OU	27.15	15.68	42.43
B / C	1.51	1.01	2.08
B / OU	13.69	7.73	21.78
C / OU	9.08	5.16	14.42

Table S7: **Model results of post-hoc contrasts of pairwise differences of surrounding input across species.** H=humans, B=bonobos, G=gorillas, C=chimpanzees, OU=orangutans. HPD interval probability: 0.95.

Contrast	Odds Ratio	Lower HPD	Upper HPD
H / B	0.27	0.04	0.75
H / C	0.72	0.11	1.87
H / OU	164.11	9.55	642.17
B / C	2.66	0.36	8.27
B / OU	610.46	25.79	2478.88
C / OU	231.60	10.63	948.22

Table S8: **Model results of post-hoc contrasts of pairwise differences of surrounding input across species, controlled for vocal activity.** H=humans, B=bonobos, G=gorillas, C=chimpanzees, OU=orangutans. HPD interval probability: 0.95.

REFERENCES AND NOTES

1. J. Huttenlocher, H. Waterfall, M. Vasilyeva, J. Vevea, L. V. Hedges, Sources of variability in children's language growth. *Cogn. Psychol.* **61**, 343–365 (2010).

2. M. L. Rowe, A longitudinal investigation of the role of quantity and quality of child-directed speech in vocabulary development. *Child Dev.* **83**, 1762–1774 (2012).

3. A. Weisleder, A. Fernald, Talking to children matters: Early language experience strengthens processing and builds vocabulary. *Psychol. Sci.* **24**, 2143–2152 (2013).

4. A. Fernald, T. Taeschner, J. Dunn, M. Papousek, B. De Boysson-Bardies, I. Fukui, A cross-language study of prosodic modifications in mothers' and fathers' speech to preverbal infants. *J. Child Lang.* **16**, 477–501 (1989).

5. C. Cox, C. Bergmann, E. Fowler, T. Keren-Portnoy, A. Roepstorff, G. Bryant, R. Fusaroli, A systematic review and Bayesian meta-analysis of the acoustic features of infant-directed speech. *Nat. Hum. Behav.* **7**, 114–133 (2023).

6. M. Soderstrom, Beyond babytalk: Re-evaluating the nature and content of speech input to preverbal infants. *Dev. Rev.* **27**, 501–532 (2007).

7. R. M. Golinkoff, D. D. Can, M. Soderstrom, K. Hirsh-Pasek, Baby talk to me: The social context of infant-directed speech and its effects on early language acquisition. *Curr. Dir. Psychol. Sci.* **24**, 339–344 (2015).

8. A. S. Holzrichter, R. P. Meier, Child-directed signing in American sign language, in *Language Acquisition by Eye* (Psychology Press), pp. 25–40 (1999).

9. J. M. Iverson, O. Capirci, E. Longobardi, M. C. Caselli, Gesturing in mother-child interactions. *Cogn. Dev.* **14**, 57–75 (1999).

10. M. Spinelli, M. Fasolo, J. Mesman, Does prosody make the difference? A meta-analysis on relations between prosodic aspects of infant-directed speech and infant outcomes. *Dev. Rev.* **44**, 1–18 (2017).

11. A. Henninga, T. Strianoa, E. V. M. Lieven, Maternal speech to infants at 1 and 3 months of age. *Infant Behav. Dev.* **28**, 519–536 (2005).

12. B. Ambridge, E. Kidd, C. F. Rowland, A. L. Theakston, The ubiquity of frequency effects in first language acquisition. *J. Child Lang.* **42**, 239–273 (2015).

13. A. Martin, Y. Igarashi, N. Jincho, R. Mazuka, Utterances in infant-directed speech are shorter, not slower. *Cognition* **156**, 52–59 (2016).

14. A. Fernald, Four-month-old infants prefer to listen to motherese. *Infant Behav. Dev.* **8**, 181–195 (1985).

15. The ManyBabies Consortium, Quantifying sources of variability in infancy research using the infant-directed-speech preference. *Adv. Methods Pract. Psychol. Sci.* **3**, 24–52 (2020).

16. J. Y. Song, K. Demuth, J. Morgan, Effects of the acoustic properties of infant-directed speech on infant word recognition. *J. Acoust. Soc. Am.* **128**, 389–400 (2010).

17. Z. O. Weizman, C. E. Snow, Lexical output as related to children's vocabulary acquisition: Effects of sophisticated exposure and support for meaning. *Dev. Psychol.* **37**, 265–279 (2001).

18. G. Csibra, G. Gergely, Natural pedagogy. *Trends Cogn. Sci.* **13**, 148–153 (2009).

19. C. Pye, Quiché Mayan speech to children. *J. Child Lang.* **13**, 85–100 (1986).

20. P. Brown, The Cultural Organization of Attention in *The Handbook of Language Socialization*, A. Duranti, E. Ochs, B. B. Schieffelin, Eds. (John Benjamins B.V., 2011), pp. 29–55.

21. L. A. Shneidman, S. Goldin-Meadow, Language input and acquisition in a Mayan village: How important is directed speech? *Dev. Sci.* **15**, 659–673 (2012).

22. A. Cristia, M. Gurven, J. Stieglitz, Child-directed speech is infrequent in a forager-farmer population: A time allocation study. *Child Dev.* **90**, 759–773 (2019).

23. M. Casillas, P. Brown, S. C. Levinson, Early language experience in a Tseltal Mayan village. *Child Dev.* **91**, 1819–1835 (2020).

24. E. K. McClay, S. Cebioglu, T. Broesch, H. H. Yeung, Rethinking the phonetics of baby-talk: Differences across Canada and Vanuatu in the articulation of mothers' speech to infants. *Dev. Sci.* **25**, e13180 (2022).

25. R. Foushee, M. Srinivasan, Infants who are rarely spoken to nevertheless understand many words. *Proc. Natl. Acad. Sci. U.S.A.* **121**, e2311425121 (2024).

26. M. Casillas, P. Brown, S. C. Levinson, Early language experience in a Papuan community. *J. Child Lang.* **48**, 792–814 (2020).

27. G. Loukatou, C. Scaff, K. Demuth, A. Cristia, N. Havron, Child-directed and overheard input from different speakers in two distinct cultures. *J. Child Lang.* **49**, 1173–1192 (2022).

28. J. Schick, C. Fryns, F. Wegdell, M. Laporte, K. Zuberbühler, C. P. van Schaik, S. W. Townsend, S. Stoll, The function and evolution of child-directed communication. *PLoS Biol.* **20**, e3001630 (2022).

29. M. Fröhlich, R. M. Wittig, S. Pika, Should I stay or should I go? Initiation of joint travel in mother–infant dyads of two chimpanzee communities in the wild. *Anim. Cogn.* **19**, 483–500 (2016).

30. D. K. Oller, U. Griebel, S. N. Iyer, Y. Jhang, A. S. Warlaumont, R. Dale, J. Call, Language Origins Viewed in Spontaneous and Interactive Vocal Rates of Human and Bonobo Infants. *Front. Psychol.* **10**, 729 (2019).

31. J. Altmann, Observational study of behavior: Sampling methods. *Behaviour* **49**, 227–267 (1974).

32. R. M. Seyfarth, D. L. Cheney, Production, usage, and comprehension in animal vocalizations. *Brain Lang.* **115**, 92–100 (2010).

33. A. Fernald, T. Simon, Expanded intonation contours in mothers' speech to newborns. *Dev. Psychol.* **20**, 104–113 (1984).

34. E. M. Luef, K. Liebal, Infant-directed communication in lowland gorillas (*Gorilla gorilla*): Do older animals scaffold communicative competence in infants? *Am. J. Primatol.* **74**, 841–852 (2012).

35. M. Fröhlich, G. Müller, C. Zeiträg, R. M. Wittig, S. Pika, Gestural development of chimpanzees in the wild: The impact of interactional experience. *Anim. Behav.* **134**, 271–282 (2017).

36. A. Knox, J. Markx, E. How, A. Azis, C. Hobaiter, F. J. van Veen, H. Morrogh-Bernard, Gesture use in communication between mothers and offspring in wild orang-utans (*Pongo pygmaeus wurmbii*) from the Sabangau peat-swamp forest, Borneo. *Intl. J. Primatol.* **40**, 393–416 (2019).

37. M. Halina, F. Rossano, M. Tomasello, The ontogenetic ritualization of bonobo gestures. *Anim. Cogn.* **16**, 653–666 (2013).

38. P. Szenczi, O. Bánszegi, A. Urrutia, T. Faragó, R. Hudson, Mother–offspring recognition in the domestic cat: Kittens recognize their own mother's call. *Dev. Psychobiol.* **58**, 568–577 (2016).

39. J. P. Balcombe, G. F. McCracken, Vocal recognition in Mexican free-tailed bats: do pups recognize mothers? *Anim. Behav.* **43**, 79–87 (1992).

40. B. M. Weiss, F. Ladich, P. Spong, H. Symonds, Vocal behavior of resident killer whale matrilines with newborn calves: The role of family signatures. *J. Acoust. Soc. Am.* **119**, 627–635 (2006).

41. D. Y. Takahashi, A. R. Fenley, Y. Teramoto, D. Z. Narayanan, J. I. Borjon, P. Holmes, A. A. Ghazanfar, The developmental dynamics of marmoset monkey vocal production. *Science* **349**, 734–738 (2015).

42. D. Y. Takahashi, D. A. Liao, A. A. Ghazanfar, Vocal learning via social reinforcement by infant marmoset monkeys. *Curr. Biol.* **27**, 1844–1852.e6 (2017).

43. D. Y. Takahashi, A. R. Fenley, A. A. Ghazanfar, Early development of turn-taking with parents shapes vocal acoustics in infant marmoset monkeys. *Philos. Trans. R. Soc. B. Biol. Sci.* **371**, 20150370 (2016).

44. A. A. Fernandez, M. Knörnschild, Pup directed vocalizations of adult females and males in a vocal learning bat. *Front. Ecol. Evol.* **8**, 265 (2020).

45. L. S. Sayigh, N. El Haddad, P. L. Tyack, V. M. Janik, R. S. Wells, F. H. Jensen, Bottlenose dolphin mothers modify signature whistles in the presence of their own calves. *Proc. Natl. Acad. Sci. U.S.A.* **120**, e2300262120 (2023).

46. V. M. Janik, M. Knörnschild, Vocal production learning in mammals revisited. *Philos. Trans. R. Soc. B Biol. Sci.* **376**, 20200244 (2021).

47. A. M. Ashbury, E. P. Willems, S. S. Utami Atmoko, F. Saputra, C. P. van Schaik, M. A. van Noordwijk, Home range establishment and the mechanisms of philopatry among female Bornean orangutans (*Pongo pygmaeus wurmbii*) at Tuanan. *Behav. Ecol. Sociobiol.* **74**, 42 (2020).

48. P. Floor, N. Akhtar, Can 18-month-old infants learn words by listening in on conversations? *Inf. Dent.* **9**, 327–339 (2006).

49. A. Fitch, A. M. Lieberman, R. J. Luyster, S. Arunachalam, Toddlers' word learning through overhearing: Others' attention matters. *J. Exp. Child Psychol.* **193**, 104793 (2020).

50. J. Schick, M. M. Daum, S. Stoll, Input to the language learning infant: The impact of other children (2024). 10.31219/osf.io/e547z.

51. C. J. Charvet, Cutting across structural and transcriptomic scales translates time across the lifespan in humans and chimpanzees. *Proc. Biol. Sci.* **288**, 20202987 (2021).

52. T. Breuer, M. B.-N. Hockemba, C. Olejniczak, R. J. Parnell, E. J. Stokes, Physical maturation, life-history classes and age estimates of free-ranging Western gorillas - Insights from Mbeli Bai, Republic of Congo. *Am. J. Primatol.* **71**, 106–119 (2009).

53. S. M. Lee, C. M. Murray, E. V. Lonsdorf, B. Fruth, M. A. Stanton, J. Nichols, G. Hohmann, Wild bonobo and chimpanzee females exhibit broadly similar patterns of behavioral maturation but some evidence for divergence. *Am. J. Phys. Anthropol.* **171**, 100–109 (2020).

54. M. A. van Noordwijk, C. P. van Schaik, Development of ecological competence in Sumatran orangutans. *Am. J. Phys. Anthropol.* **127**, 79–94 (2005).

55. R. S. Mendonça, T. Kanamori, N. Kuze, M. Hayashi, H. Bernard, T. Matsuzawa, Development and behavior of wild infant-juvenile East Bornean orangutans (*Pongo pygmaeus morio*) in Danum Valley. *Primates* **58**, 211–224 (2017).

56. M. A. van Noordwijk, S. S. U. Atmoko, C. D. Knott, N. Kuze, H. C. Morrogh-Bernard, F. Oram, C. Schuppli, C. P. van Schaik, E. P. Willems, The slow ape: High infant survival and long interbirth intervals in wild orangutans. *J. Hum. Evol.* **125**, 38–49 (2018).

57. M. Surbeck, S. Coxe, A. L. Lokasola, Lonoa: The establishment of a permanent field site for behavioural research on Bonobos in the Kokolopori Bonobo Reserve. *Pan Africa News* **24**, 13–15 (2017).

58. V. Reynolds, *The Chimpanzees of the Budongo Forest: Ecology, Behaviour and Conservation* (OUP Oxford, 2005).

59. M. A. van Noordwijk, L. R. LaBarge, J. A. Kunz, A. M. Marzec, B. Spillmann, C. Ackermann, P. Rianti, E. R. Vogel, S. S. U. Atmoko, M. Kruetzen, C. van Schaik, Reproductive success of Bornean orangutan males: Scattered in time but clustered in space. *Behav. Ecol. Sociobiol.* **77**, 134 (2023).

60. P. Valenzuela, *Transitivity in Shipibo-Konibo grammar: A typologically oriented study*, (dissertation, University of Oregon, Eugene, OR) (2003).

61. B. Hellwig, D. Jung, Events of caused accompanied motion in Qaqet and Dene Suline child language corpora. Caused Accompanied Motion p. 397 (2022).

62. Stoll S. Lieven E. Banjade G. Bhatta T. N. Gaenszle M. Paudyal N. P. Rai M. Rai N. K. Rai I. P. Zakharko T. Audiovisual corpus on the acquisition of Chintang by six children 2015

63. X. A. Harrison, A comparison of observation-level random effect and Beta-binomial models for modelling overdispersion in binomial data in ecology & evolution. *PeerJ* **3**, e1114 (2015).

64. R Core Team R: A language and environment for statistical computing (Computer software manual) (2022) https://R-project.org

65. P.-C. Bürkner, Bayesian item response modeling in R with brms and Stan. *J. Stat. Softw.* **100**, 1–54 (2021).

66. B. Carpenter, A. Gelman, M. D. Hoffman, D. Lee, B. Goodrich, M. Betancourt, M. A. Brubaker, J. Guo, P. Li, A. Riddell, Stan: A probabilistic programming language. *J. Stat. Softw.* **76**, 1 (2017).

67. A. Gelman, B. Goodrich, J. Gabry, A. Vehtari, R-squared for Bayesian regression models. *Am. Stat.* **73**, 307–309 (2019).

68. C. Hobaiter, R. W. Byrne, The meanings of chimpanzee gestures. *Curr. Biol.* **24**, 1596–1600 (2014).

69. M. Davila-Ross, B. Allcock, C. Thomas, K. A. Bard, Aping expressions? Chimpanzees produce distinct laugh types when responding to laughter of others. *Emotion* **11**, 1013–1020 (2011).

70. S. L. Winkler, G. A. Bryant, Play vocalisations and human laughter: A comparative review. *Bioacoustics* **30**, 499–526 (2021).

71. S. A. Wich, M. Krützen, A. R. Lameira, A. Nater, N. Arora, M. L. Bastian, E. Meulman, H. C. Morrogh-Bernard, S. S. U. Atmoko, J. Pamungkas, D. Perwitasari-Farajallah, M. E. Hardus, M. van Noordwijk, C. P. van Schaik, Call cultures in orang-utans? *PLOS ONE* **7**, e36180 (2012).

72. M. Gamer, J. Lemon, I. F. P. Singh, *irr: Various Coefficients of Interrater Reliability and Agreement* (2019), https://CRAN.R-project.org/package=irr, r package version 0.84.1.

73. A. B. Kaufman, R. Rosenthal, Can you believe my eyes? The importance of interobserver reliability statistics in observations of animal behaviour. *Anim. Behav.* **78**, 1487–1491 (2009).
